# Deletion of *Phd2* in Myeloid Lineage Attenuates Hypertensive Cardiovascular Remodeling

**DOI:** 10.1161/JAHA.113.000178

**Published:** 2013-06-21

**Authors:** Jiro Ikeda, Toshihiro Ichiki, Hirohide Matsuura, Eriko Inoue, Junji Kishimoto, Aya Watanabe, Chikahiro Sankoda, Shiro Kitamoto, Tomotake Tokunou, Kotaro Takeda, Guo‐Hua Fong, Kenji Sunagawa

**Affiliations:** 1 Department of Cardiovascular Medicine, Kyushu University Graduate School of Medical Sciences, Fukuoka, Japan (J.I., T.I., H.M., E.I., A.W., C.S., S.K., T.T., K.S.); 2 Department of Advanced Therapeutics for Cardiovascular Diseases, Kyushu University Graduate School of Medical Sciences, Fukuoka, Japan (T.I., S.K.); 3 Center for Clinical and Translational Research, Kyushu University Hospital, Fukuoka, Japan (J.K.); 4 Department of Cell Biology, Center for Vascular Biology, University of Connecticut Health Center, Farmington, CT (K.T., G.H.F.)

**Keywords:** fibrosis, hypertrophy, hypoxia, macrophages, migration

## Abstract

**Background:**

Hypertension induces cardiovascular hypertrophy and fibrosis. Infiltrated macrophages are critically involved in this process. We recently reported that inhibition of prolyl hydroxylase domain protein 2 (PHD2), which hydroxylates the proline residues of hypoxia‐inducible factor‐α (HIF‐α) and thereby induces HIF‐α degradation, suppressed inflammatory responses in macrophages. We examined whether myeloid‐specific *Phd2* deletion affects hypertension‐induced cardiovascular remodeling.

**Methods and Results:**

Myeloid‐specific PHD2‐deficient mice (MyPHD2KO) were generated by crossing *Phd2*‐floxed mice with LysM‐Cre transgenic mice, resulting in the accumulation of HIF‐1α and HIF‐2α in macrophage. Eight‐ to ten‐week‐old mice were given *N*^G^‐nitro‐L‐arginine methyl ester (L‐NAME), a nitric oxide synthase inhibitor, and Angiotensin II (Ang II) infusion. L‐NAME/Ang II comparably increased systolic blood pressure in control and MyPHD2KO mice. However, MyPHD2KO mice showed less aortic medial and adventitial thickening, and macrophage infiltration. Cardiac interstitial fibrosis and myocyte hypertrophy were also significantly ameliorated in MyPHD2KO mice. *Transforming growth factor*‐β and *collagen* expression were decreased in the aorta and heart from MyPHD2KO mice. Echocardiographic analysis showed that left ventricular hypertrophy and reduced ejection fraction induced by L‐NAME/Ang II treatment in control mice were not observed in MyPHD2KO mice. Administration of digoxin that inhibits HIF‐α synthesis to L‐NAME/Ang II‐treated MyPHD2KO mice reversed these beneficial features.

**Conclusions:**

*Phd2* deletion in myeloid lineage attenuates hypertensive cardiovascular hypertrophy and fibrosis, which may be mediated by decreased inflammation‐ and fibrosis‐associated gene expression in macrophages. PHD2 in myeloid lineage plays a critical role in hypertensive cardiovascular remodeling.

## Introduction

Hypertension induces cardiovascular remodeling such as hypertrophy and fibrosis. It is generally accepted that the renin‐angiotensin system (RAS) and nitric oxide (NO) system play critical roles in this process, and increased RAS activity and reduced NO level are common features observed in patients with essential hypertension.^1^ Hypertension also induces inflammation of the cardiovascular system. Infiltrated inflammatory cells including macrophages play an important role in cardiovascular remodeling.^2^ It has been reported that blockade of monocyte chemoattractant protein‐1 (MCP‐1)/chemokine receptor 2 pathway prevents vascular inflammation and arteriosclerosis in a hypertensive rat model induced by chronic inhibition of NO synthesis.^3^ Cytokines derived from macrophages such as tumor necrosis factor (TNF)‐α and MCP‐1 are involved in the development of cardiac hypertrophy.^4–5^ Macrophages also contribute to the pathogenesis of fibrosis. Transforming growth factor‐β (TGF‐β) derived from macrophages promotes fibroblast differentiation into myofibroblasts that regulate deposition of an extracellular matrix and induce collagen synthesis in myofibroblasts.^6^ Tissue hypoxia also plays a critical role in cardiovascular remodeling. Hypoxia induces matrix production such as type 1 collagen and vascular smooth muscle cell (SMC) proliferation.^7–8^

Hypoxia‐inducible factor (HIF) is a key transcription factor that regulates cellular responses to hypoxia.^9^ Hypoxia‐inducible factor is composed of an O_2_‐sensitive α‐subunit and a constitutively expressed β‐subunit.^10^ Under normoxic condition, prolyl hydroxylase domain protein (PHD) hydroxylates the proline residues of HIF‐α, which induces subsequent ubiquitination and proteasomal degradation.^11^ Under hypoxic condition, PHD activity is inhibited, resulting in stabilization and accumulation of HIF‐α in the nucleus. HIF‐α and HIF‐β form a heterodimer and activate expression of target genes that regulate angiogenesis, erythropoiesis, and metabolism.^12^ There are 2 isoforms of HIF‐α designated HIF‐1α and HIF‐2α, which activate distinct target genes, and 3 isoforms for PHD (PHD1‐3) have been reported.^13^ These PHD isoforms show differential affinity for HIF‐1α and HIF‐2α and tissue distribution. It is suggested that PHD2 is mainly involved in the hypoxic response.^14^

Hypoxia inducible factor also regulates the inflammatory process. Mice lacking HIF‐1α in the myeloid lineage revealed impaired inflammatory responses.^15^ Deletion of *HIF‐1α* impairs aggregation and motility of myeloid cells. We previously showed that pharmacological inhibition of PHD or knock down of PHD2 attenuates lipopolysaccharide (LPS)‐induced TNF‐α up‐regulation in macrophages.^16^ These studies prompted us to examine whether myeloid‐specific *Phd2* deletion affects hypertensive vascular and cardiac remodeling.

## Materials and Methods

### Materials

Dulbecco's Modified Eagle Medium (DMEM) was purchased from GIBCO‐BRL, Invitrogen Co. Fetal bovine serum (FBS) was purchased from SAFC Biosciences Inc. Thioglycollate was purchased from Becton Dickinson and Co. *N*^G^‐nitro‐L‐arginine methyl ester (L‐NAME), LPS from *Escherichia coli*, recombinant MCP‐1, digoxin, and a monoclonal anti‐α‐tubulin antibody were purchased from Sigma‐Aldrich Co. Complete Protease Inhibitor Cocktail solution was purchased from Roche Applied Science. An antibody against PHD2 was custom‐made by Maine Biotechnology Services in Wistar rats against a C‐terminal peptide (EKGVRVELKPNSVSKDV), and supernatants from fused hybridomas were used without clonal purification. An antibody against Mac‐2 was purchased from Cedarlane Laboratories. Polyclonal antibodies against HIF‐1α and HIF‐2α were purchased from Novus Biologicals, Inc. An antibody against Histone H3 was purchased from Cell Signaling Technology, Inc. An antibody against TGF‐β was purchased from Chemicon International. Horseradish peroxidase‐conjugated secondary antibodies were purchased from Vector Laboratories, Inc. Angiotensin (Ang) II was purchased from Peptide Institute Inc. Other chemical reagents were purchased from Wako Pure Chemical Industries, Ltd unless otherwise stated.

### Animals and Animal Experiment

All procedures were approved by Animal Care and Use Committee, Kyushu University and conducted in accordance with the institutional guidelines. LysM‐Cre mice that expression of *Cre recombinase* is driven by *lysozyme M* (*LysM*) gene promoter were purchased from Riken Bio Resource Center. Myeloid‐specific PHD2‐deficient mice (MyPHD2KO) on a C57BL/6 background were generated by crossing heterozygous *Phd2*‐floxed mice (*Phd2*^*f/+*^) with LysM‐Cre mice. Cohort of control (*Phd2*^*f/f*^) and MyPHD2KO (*Phd2*^*f/f*^/LysM‐Cre) mice were generated by crossing *Phd2*^*f/f*^ and MyPHD2KO mice. Eight‐ to ten‐week‐old mice were given 30 mg/kg per day of L‐NAME dissolved in 0.9% NaCl in drinking water for 14 days. Angiotensin II (0.8 mg/kg per day) was infused subcutaneously via an ALZET osmotic mini‐pump (Durect Co) for the last 7 days. Digoxin (2 mg/kg per day) was injected intraperitoneally every second day from 7 days before starting L‐NAME administration and continued until the end of experiment.^17^ Mice were fed a normal chow. The following 5 groups were examined: (1) Control, (2) MyPHD2KO, (3) Control+L‐NAME/Ang II, (4) MyPHD2KO+L‐NAME/Ang II, and (5) MyPHD2KO+L‐NAME/Ang II+Digoxin. Heart rate (HR) and systolic blood pressure (SBP) were measured using tail‐cuff method (BP‐98A, Softron Co) in conscious mice. Transthoracic echocardiographic analysis was performed using the VEVO 2100 Ultrasound System with 40 MHz MS550D transducer (Visual Sonics) under anesthesia (1.5% isoflurane). Wall thickness of interventricular septum and posterior wall, left ventricular (LV) end‐systolic dimension (ESD) and end‐diastolic dimension (EDD) were determined by M‐mode echocardiography. Data were average of 3 to 5 beats. Fractional shortening (FS) and ejection fraction (EF) were calculated based on the following formulas. FS=(LVEDD−LVESD)/LVEDD×100 (%), end‐diastolic volume (EDV)=7× LVEDD^3^/(2.4+LVEDD), end‐systolic volume (ESV)=7× LVESD^3^/(2.4+LVESD) and EF=(EDV−ESV)/EDV×100 (%).

### Complete Blood Cell Count

Whole blood was collected from tail vein into tubes with ethylenediaminetetraacetic acid. The number of white blood cell, red blood cell and platelet, and hemoglobin concentration were measured by Hematology analyzer MEK‐6450 Celltac α (Nihon Kohden).

### Isolation of Peritoneal Macrophages and Bone Marrow‐Derived Macrophages

Mice were intraperitoneally injected with 2.0 mL of 3% thioglycollate. Four days later, the peritoneal cavity was lavaged with 6 mL PBS to retrieve infiltrated cells. After centrifugation (1000 rpm, 5 minutes, 4°C), the pelleted cells were resuspended in DMEM containing 10% FBS, seeded in cell culture dish and incubated at 37°C, with 5% CO_2_ overnight. Nonadherent cells were removed by washing with PBS and adherent cells were used as peritoneal macrophages (PMs). Bone marrow cells were isolated from femurs and tibias and centrifuged (1000 rpm, 5 minutes, 4°C). The pelleted cells were resuspended in DMEM supplemented with 10% FBS and 30% L929 conditioned medium as a source of macrophage‐colony stimulating factor for 7 days and used as bone marrow‐derived macrophage (BMDMs).^18^

### Histological Analysis

At the end of experiment, mice were euthanized under CO_2_ inhalation. The hearts and aortas were removed and frozen at −80°C in O.C.T. compound (Sakura Finetek), cut into 6‐μm sections, and fixed with 4% paraformaldehyde. The frozen sections of aorta were stained with Sirius red solutions and the heart sections were stained with Masson Trichrome solutions. The stained sections were scanned using a fluorescence microscope (BZ‐9000, Keyence). The aortic medial area, adventitial area, and the interstitial fibrotic area of the heart were quantified using image processing software (ImageJ). The interstitial fibrotic area of the heart was measured as a percentage of the total area of each section. For measuring the cross‐sectional area of myocyte, heart sections were stained with wheat germ agglutinin (WGA)‐Alexa Fluor 488 conjugate (Invitrogen) and the size of myocytes was measured by using hybrid cell count software (BZ‐H1C, Keyence). Data from at least 300 cells were produced per slide. Paraffin‐embedded tissue sections were used for Mac‐2 immunohistochemistry. For analyzing immunohistochemical staining of aorta and heart for Mac‐2, Mac‐2‐positive cells per section were counted. Three sections per mouse were measured for all histological analyses. An independent investigator blind to the treatment or genotype of the mice counted the Mac‐2‐positive cells and measured medial area and fibrotic area.

### Western Blot Analysis

Western blot analysis was performed by a conventional method as described previously.^19^ To prepare nuclear protein, PMs were suspended in a buffer containing 10 mmol/L HEPES‐KOH (pH 7.9), 1.5 mmol/L MgCl_2_, 10 mmol/L KCl, 0.5 mmol/L DTT, 0.2 mmol/L phenylmethylsulfonyl fluoride, 0.2 mmol/L CoCl_2_, 1× complete Protease Inhibitor Cocktail solution (Roche Applied Science) and 0.6% NP‐40. Nuclei were pelleted by centrifugation (1000 *g*) and nuclear proteins were extracted with a buffer containing 20 mmol/L HEPES‐KOH, 1.5 mmol/L MgCl_2_, 420 mmol/L NaCl, 0.5 mmol/L DTT, 0.2 mmol/L CoCl_2_, 25% glycerol, and 1× Complete Protease Inhibitor Cocktail solution. Nuclear or total cell extracts were cleared by centrifugation and used for Western blot analysis. Expression level of HIF‐1α and HIF‐2α in PMs was indicated as a ratio of HIF‐1α and HIF‐2α to Histone H3.

### Real‐Time Reverse Transcription‐Quantitative PCR Analysis

The aortas and hearts were removed, minced into small pieces, and homogenized in ISOGEN (Nippon Gene Co, Ltd) on ice. Total RNA was extracted according to the manufacturer's protocol. Total RNA from PMs or BMDMs was extracted with High Pure RNA isolation kit (Roche Applied Science). One microgram of total RNA was reverse‐transcribed with ReverTra Ace qPCR RT Kit (TOYOBO Co, Ltd). Real‐Time Reverse Transcription‐Quantitative PCR (RT‐qPCR) was performed using THUNDERBIRD SYBR qPCR mix (TOYOBO) and the ABI PRISM 7500 Sequence Detection System (Applied Biosystems). Relative expression levels were determined by standard curve method. Expression of *Phd1, Phd2, Phd3, Tnfa, Il6, Il1b, Rantes, Mcp1, iNOS, Arg1, Fizz1, Mrc1, Pdgfb, Tgfb, Ctgf, F4/80, Col1a2, Col3a1, Anp, and Cd177* was presented as the relative mRNA level to that of *Hprt* mRNA. The sequences of primers and abbreviations are summarized in Table 1.

**Table 1.** List of the Primer Sequences Used for RT‐qPCR

mRNA	Forward Primer	Reverse Primer
*Anp*	5'‐TGACAGGATTGGAGCCCAGA‐3'	5'‐GACACACCACAAGGGCTTAGGA‐3'
*Arg1*	5'‐AGCTCTGGGAATCTGCATGG‐3'	5'‐ATGTACACGATGTCTTTGGCAGATA‐3'
*Cd177*	5'‐CACTGACTCTGTCACCTGCCCTA‐3'	5'‐TTTGGAGTCACCCAGTAAAGGTTTG‐3'
*Col1a2*	5'‐CTTCTGCAGGGTTCCAACGA‐3'	5'‐CAGCACCACCAATGTCCAGAG‐3'
*Col3a1*	5'‐CAGGCCAGTGGCAATGTAAAGA‐3'	5'‐CAGCACCACCAATGTCCAGAG‐3'
*Ctgf*	5'‐ACCCGAGTTACCAATGACAATACC‐3'	5'‐CGCTGAATCGAAAGCCCTGTA‐3'
*F4/80*	5'‐GAGATTGTGGAAGCATCCGAGAC‐3'	5'‐GATGACTGTACCCACATGGCTGA‐3'
*Fizz1*	5'‐TCCAGCTGATGGTCCCAGTG‐3'	5'‐GAGGCCCATCTGTTCATAGTCTTG‐3'
*Hprt*	5'‐TTGTTGTTGGATATGCCCTTGACTA‐3'	5'‐AGGCAGATGGCCACAGGACTA‐3'
*iNOS*	5'‐CAAGCTGAACTTGAGCGAGGA‐3'	5'‐TTTACTCAGTGCCAGAAGCTGGA‐3'
*Il1b*	5'‐TCCAGGATGAGGACATGAGCAC‐3'	5'‐GAACGTCACACACCAGCAGGTTA‐3'
*Il6*	5'‐CCACTTCACAAGTCGGAGGCTTA‐3'	5'‐GCAAGTGCATCATCGTTGTTCATAC‐3'
*Mcp1*	5'‐GCATCCACGTGTTGGCTCA‐3'	5'‐CTCCAGCCTACTCATTGGGATCA‐3'
*Mrc1*	5'‐AGCTTCATCTTCGGGCCTTTG‐3'	5'‐GGTGACCACTCCTGCTGCTTTAG‐3'
*Pdgfb*	5'‐CAAAGGCAAGCACCGAAAGTTTA‐3'	5'‐CCGAATCAGGCATCGAGACA‐3'
*Phd1*	5'‐CATCAATGGGCGCACCA‐3'	5'‐GATTGTCAACATGCCTCACGTAC‐3'
*Phd2*	5'‐TAAACGGCCGAACGAAAGC‐3'	5'‐GGGTTATCAACGTGACGGACA‐3'
*Phd3*	5'‐CTATGTCAAGGAGCGGTCCAA‐3'	5'‐GTCCACATGGCGAACATAACC‐3'
*Rantes*	5'‐ACCAGCAGCAAGTGCTCCAA‐3'	5'‐TGGCTAGGACTAGAGCAAGCAATG‐3'
*Tgfb*	5'‐GTGTGGAGCAACATGTGGAACTCTA‐3'	5'‐CTCATTGCCTTGCGTGTTTGATA‐3'
*Tnfa*	5'‐AAGCCTGTAGCCCACGTCGTA‐3'	5'‐GGCACCACTAGTTGGTTGTCTTTG‐3'

RT‐qPCR indicates real‐time reverse transcription‐quantitative polymerase chain reaction; Anp, atrial natriuretic peptide; Arg, arginase; Col, collagen; Ctgf, connective tissue growth factor; Fizz, found in inflammatory zone; Hprt, hypoxanthine phosphoribosyl‐transferase; iNOS, inducible nitric oxide synthase; Il, interleukin; Mcp, monocyte chemoattractant protein; Mrc, mannose receptor c; Pdgf, platelet‐derived growth factor; Phd, prolyl hydroxylase domain protein; Rantes, regulated on activation normal T cell expressed and secreted; Tgf, transforming growth factor; Tnf, tumor necrosis factor.

### Chemotaxis Assay

A chemotaxis assay was performed with 96‐well ChemoTx System (Neuro Probe Inc) using isolated PMs in accordance with the manufacturer's instruction. Peritoneal macrophages (2×10^6^ cells/mL) in DMEM containing 0.1% BSA were loaded onto the upper wells. The lower wells separated by polycarbonate membrane with 8 μm pores were filled with the same medium containing MCP‐1 (0, 10, 25 ng/mL). After incubation for 16 hours at 37°C, he number of migrated cells per field on the lower surface of the membrane were counted after staining with Diff‐Quik (Sysmex). Experiments were performed in duplicate and were repeated at least 3 times.

### NF‐ κB Transcriptional Activity

Isolated PMs were seeded in 96 well plates (2000 cells/well) and infected with Cignal Lenti NF‐κB Reporter (SA Bioscience), which was delivered as ready‐to‐transduce lentiviral particles expressing the firefly luciferase gene under the control of a minimal cytomegalovirus promoter and tandem repeats of NF‐κB transcriptional response element, with a multiplicity of infection of 40. After 18 hours of infection, the medium was changed and cells were incubated for 24 hours. Transfected macrophages were stimulated with LPS (100 ng/mL) for 24 hours, cell lysate was prepared and luciferase activity was measured in Lumat LB9501 (Berthold Technologies). Luciferase activity was standardized by protein concentration measured by Micro BCA Protein Assay Kit (Pierce Biotechnology).

### Laser Capture Microdissection

Fresh frozen heart sections cut into 7‐μm sections were fixed in RNase free ethyl acetate and stained with 0.05% toluidine blue solution. The region of cardiac tissue area without visible infiltrating cells was dissected using LMD6000 system (Leica Microsystems) and collected in the cap of collection tube containing RLT buffer of RNeasy Mini Kit (Qiagen). Six sections with a total of 1.0×10^6^ μm^2^ were collected from each mouse. Total RNA was extracted in accordance with the manufacturer's instruction and 100 ng of total RNA was reverse‐transcribed and RT‐qPCR was performed as described.

### Statistical Analysis

The data were checked for normality using Shapiro‐Wilk test. A *t* test was used for pair‐wise comparisons. One‐ and 2‐factor ANOVA were used to compare >2 groups, and, if significant, pairwise comparisons were performed using Fisher's post‐hoc test to account for multiple comparisons. Data are shown as mean±SEM. Values of *P*<0.05 were considered to be statistically significant.

## Results

### Generation of Myeloid‐Specific PHD2‐Deficient Mice

**Figure 1.**
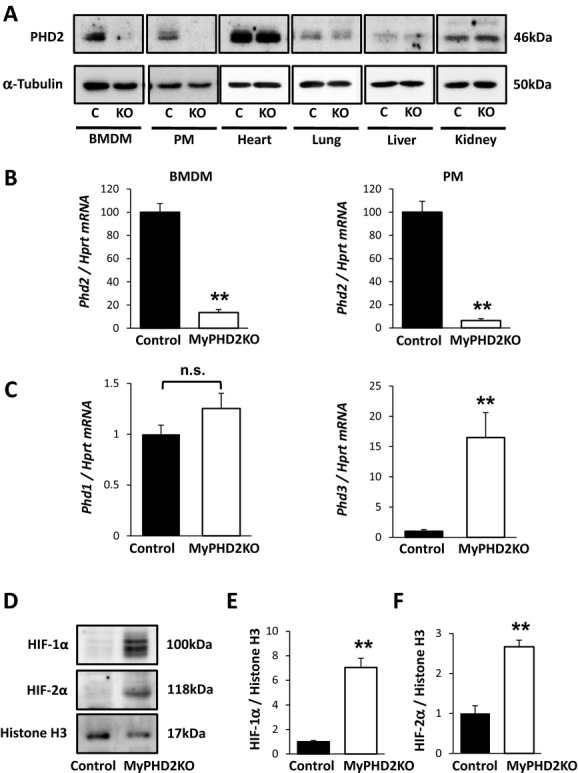
Suppression of PHD2 protein and mRNA expression in MyPHD2KO mice. A, Western blot for PHD2 in bone marrow derived macrophages (BMDMs), peritoneal macrophages (PMs), heart, lung, liver, and kidney in control and MyPHD2KO mice is shown. The same results were obtained in other 2 independent experiments. B, *Phd2 *mRNA expression in BMDMs and PMs was measured by RT‐qPCR. **P<0.01 vs Control. n=8. C, *Phd1* and *Phd3 *mRNA expression in PMs were measured by RT‐qPCR. **P<0.01 vs Control. n=8. D, Western blot for HIF‐1α and HIF‐2α in nuclear protein of PMs is shown. E and F, The bar graphs indicate the expression ratio of HIF‐1α and HIF‐2α to Histone H3, respectively. **P<0.01 vs Control. n=3. PHD2 indicates prolyl hydroxylase domain protein 2; C, Control; KO, MyPHD2KO; RT‐qPCR, real‐time reverse transcription‐quantitative polymerase chain reaction; HIF, hypoxia‐inducible factor; Hprt, hypoxanthine phosphoribosyl‐transferase.

PHD2 expression in various organs from control and MyPHD2KO mice were examined by Western blot analysis (Figure 1A). PHD2 protein was not detected in BMDMs and PMs from MyPHD2KO mice. *Phd2* mRNA expression determined by RT‐qPCR in BMDMs and PMs from MyPHD2KO mice was significantly suppressed compared with control mice (Figure 1B). The expression of *Phd2* mRNA in the aorta was comparable between control and MyPHD2KO mice (data not shown). Next we examined the expression of other isoforms of *Phd* in PMs. Although *Phd1* mRNA level was unchanged, mRNA of *Phd3*, a target gene of HIF‐1α,^20^ was significantly higher in PHD2‐deficient PMs (Figure 1C). HIF‐1α and HIF‐2α protein levels were dramatically increased in PHD2‐deficient PMs compared with control PMs (Figure 1D through 1F). Expression of HIF‐1α and HIF‐2α protein was also increased in BMDMs from MyPHD2KO mice (data not shown). No significant changes were noted on complete blood cell count between control and MyPHD2KO mice (Table 2).

**Table 2.** Complete Blood Cell Count

	Control	MyPHD2KO	Control+L/A	MyPHD2KO+L/A	MyPHD2KO+L/A+Digoxin
WBC, ×10^2^/μL	116.6±21.4	117.6±10.1	107.4±17.3	117.6±25.4	97.0±14.7
RBC, ×10^4^/μL	910.8±24.1	870.0±28.6	800.4±60.0	915.0±76.1	892.0±30.1
Hb, g/dL	12.5±0.4	12.0±0.4	11.5±0.9	12.7±1.0	12.5±0.5
PLT, ×10^4^/μL	78.7±8.4	72.8±4.1	68.5±2.5	70.1±2.1	71.8±6.3

Data are expressed as mean±SEM. n=5. L/A indicates L‐NAME+Angiotensin II; WBC, white blood cell; RBC, red blood cell; Hb, hemoglobin; PLT, platelet; SEM, standard error of the mean.

### *Phd2* Deletion Decreased the Expression of M1 Macrophage Markers in PMs

**Figure 2.**
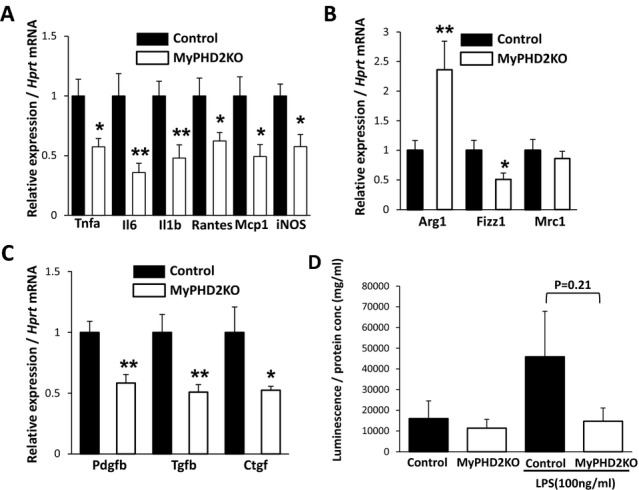
The effect of *Phd2* deletion on gene expression and NF‐κB transcriptional activity in peritoneal macrophages. mRNA expression of M1 macrophage markers (A), M2 macrophage markers (B), and fibrosis‐associated genes (C) in peritoneal macrophages (PMs) was analyzed by RT‐qPCR. *P<0.05, **P<0.01 vs Control. n=8. D, NF‐κB transcriptional activity was determined in PMs. NF‐κB‐dependent luciferase activity in isolated PMs with or without stimulation with LPS (100 ng/mL) for 24 hours was determined. Luciferase activity was standardized by protein concentration (conc). n=5. *Phd2* indicates prolyl hydroxylase domain protein 2; NF, nuclear factor; RT‐qPCR, real‐time reverse transcription‐quantitative polymerase chain reaction; LPS, lipopolysaccharide; Hprt, hypoxanthine phosphoribosyl‐transferase; Tnf, tumor necrosis factor; Il, interleukin; Rantes, regulated on activation normal T cell expressed and secreted; Mcp1, monocyte chemoattractant protein 1; iNOS, inducible nitric oxide synthase; Arg, arginase; Fizz, found in inflammatory zone; Mrc, mannose receptor c; Pdgf, platelet‐derived growth factor; Tgf, transforming growth factor; Ctgf, connective tissue growth factor.

To investigate whether PHD2 regulates macrophage polarization, we examined the expression of M1 and M2 macrophage markers in isolated PMs. The expression of *Tnfa, Il6, Il1b*,* Rantes, Mcp1, and iNOS*, markers of M1 macrophage, was significantly reduced by *Phd2* deletion (Figure 2A). However M2 macrophage markers did not show a consistent trend by *Phd2* deletion (Figure 2B). Expression of *Pdgfb*,* Tgfb* and *Ctgf* was also decreased by *Phd2* deletion (Figure 2C). These results suggest that *Phd2* deletion reduces M1 polarization without clear polarization to M2. Expression of inflammatory cytokines is regulated by nuclear factor‐κB (NF‐κB). We examined NF‐κB activity in isolated PMs. NF‐κB activity in PMs from MyPHD2KO mice was lower than that from control mice in baseline and after LPS stimulation (Figure 2D). However, the difference was not statistically significant.

### *Phd2* Deletion Suppressed Macrophage Migration

**Figure 3.**
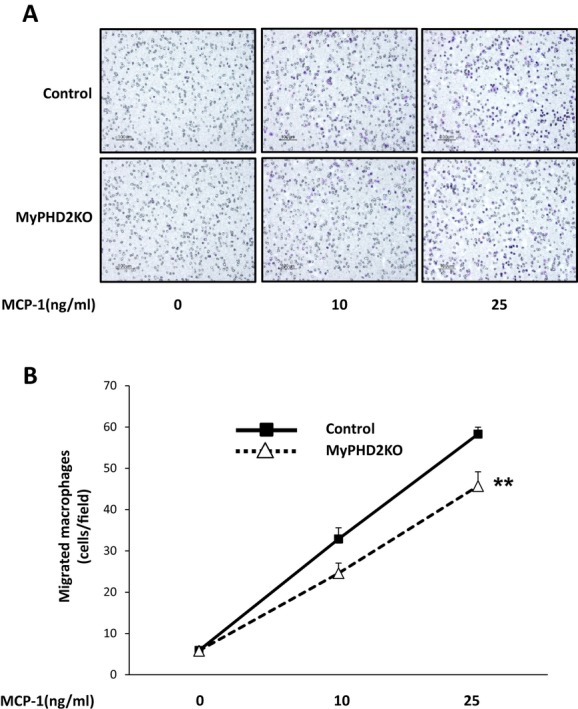
The effect of *Phd2* deletion on macrophage migration. A, Chemotactic activity of peritoneal macrophages in response to MCP‐1 (0, 10, 25 ng/mL) was analyzed. Representative photographs of lower surface of the membrane are shown. B, Summary results of the number of migrated cells/field are shown. **P<0.01 vs Control, n=5. *Phd2* indicates prolyl hydroxylase domain protein 2; MCP‐1, monocyte chemoattractant protein‐1.

We performed transwell migration assays to investigate whether *Phd2* deletion affects macrophage migration. Chemotactic activity in response to MCP‐1 was seen in both control and PHD2‐deficient macrophages. However, migration was significantly attenuated in PHD2‐deficient macrophages compared with control macrophages, suggesting that PHD2 plays an important role in MCP‐1‐induced macrophage chemotaxis (Figure 3).

### MyPHD2KO Mice Showed Less Medial Thickening Induced by High Blood Pressure

**Figure 4.**
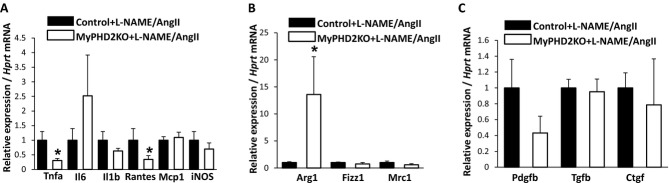
The effect of L‐NAME/Ang II treatment on macrophage polarization. mRNA expression of M1 macrophage markers (A), M2 macrophage markers (B), and fibrosis‐associated genes (C) in peritoneal macrophages from L‐NAME/Ang II‐treated control and MyPHD2KO mice was analyzed by RT‐qPCR. *P<0.05 vs Control+L‐NAME/Ang II. n=6. L‐NAME indicates *N*^G^‐nitro‐L‐arginine methyl ester; Ang II, Angiotensin II; RT‐qPCR, real‐time reverse transcription‐quantitative polymerase chain reaction; Hprt, hypoxanthine phosphoribosyl‐transferase; Tnf, tumor necrosis factor; Il, interleukin; Rantes, regulated on activation normal T cell expressed and secreted; Mcp1, monocyte chemoattractant protein 1; iNOS, inducible nitric oxide synthase; Arg, arginase; Fizz, found in inflammatory zone; Mrc, mannose receptor c; Pdgf, platelet‐derived growth factor; Tgf, transforming growth factor; Ctgf, connective tissue growth factor.

**Figure 5.**
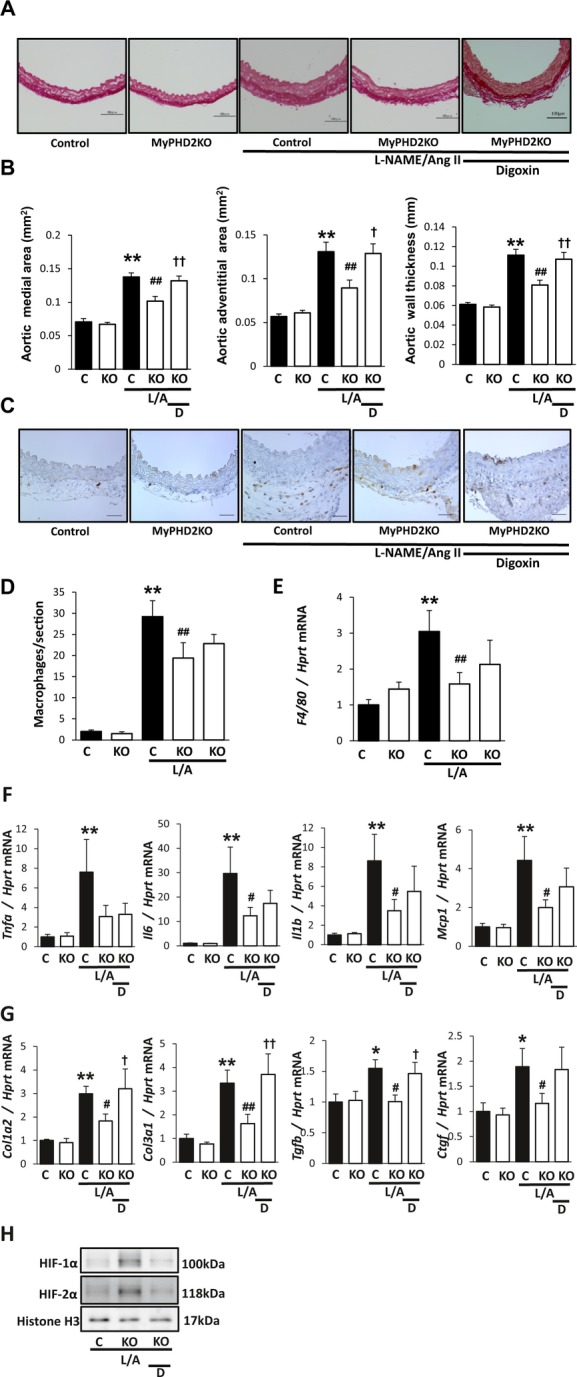
Attenuation of medial hypertrophy and adventitial fibrosis in MyPHD2KO mice. A, Representative photographs of Sirius red staining of thoracic aorta are shown. Scale bar: 100 μm. B, Summary results of medial and adventitial area and wall thickness are shown. n=14 (C), 15(KO), 15(C+L/A), 17(KO+L/A), 5(KO+L/A+D). C, Representative photographs of immunohistochemistry of thoracic aorta for Mac‐2 are shown. Scale bar: 50 μm. D, Summary results of the number of Mac‐2 positive cells/slice are shown. n=5 (C), 5 (KO), 6 (C+L/A), 6 (KO+L/A), 5 (KO+L/A+D). E, Expression of *F4/80 *mRNA in aorta was analyzed by RT‐qPCR. n=8 (C), 8 (KO), 9 (C+L/A), 10 (KO+L/A), 5 (KO+L/A+D). F, Proinflammatory gene expression in aorta was analyzed by RT‐qPCR. n=8 (C), 8 (KO), 9 (C+L/A), 10 (KO+L/A), 5 (KO+L/A+D). G, Fibrosis‐associated gene expression in aorta was analyzed by RT‐qPCR. n=8 (C), 8 (KO), 9 (C+L/A), 10 (KO+L/A), 5 (KO+L/A+D). *P<0.05, **P<0.01 vs C, #*P*<0.05, ##*P*<0.01 vs C+L/A, †*P*<0.05, ††*P*<0.01 vs KO+L/A. H, Representative Western blot analyses for HIF‐1α, HIF‐2α and Histone H3 in nuclear protein of peritoneal macrophages are shown. The same results were obtained in other independent experiments. n=3. C indicates control; KO, MyPHD2KO; L/A: L‐NAME/Ang II; D, digoxin; RT‐qPCR, real‐time reverse transcription‐quantitative polymerase chain reaction; Hprt, hypoxanthine phosphoribosyl‐transferase; Tnf, tumor necrosis factor; Il, interleukin; Mcp1, monocyte chemoattractant protein 1; Col, collagen; Tgf, transforming growth factor; Ctgf, connective tissue growth factor; HIF, hypoxia‐inducible factor.

It is generally accepted that increased RAS activity and suppression of NO levels are common features of cardiovascular diseases including essential hypertension.^1,21^ We therefore examined the effect of cotreatment with L‐NAME, a NO synthase inhibitor, and Ang II on cardiovascular remodeling in control and MyPHD2KO mice. After treatment with L‐NAME/Ang II, no significant changes were observed in complete blood cell count (Table 2), and SBP and HR were comparably increased in control and MyPHD2KO mice (Table 3). Treatment with L‐NAME/Ang II did not affect *Phd2* mRNA expression in PMs from control and MyPHD2KO mice (data not shown). Expression of *TNFα* and *RANTES* was still significantly suppressed and *Arg1* expression was increased in PMs from L‐NAME/Ang II‐treated MyPHD2KO mice compared with control mice (Figure 4). However, expression of other genes did not show significant difference. Histological examination of aorta by Sirius red staining showed no significant changes at baseline (Figure 5A). Treatment with L‐NAME/Ang II increased medial wall thickness and adventitial fibrotic area in control mice, which was attenuated in MyPHD2KO mice compared with control mice (Figure 5A and 5B). Immunohistochemical staining for Mac‐2, a macrophage marker, showed marked infiltration of macrophages into aortic wall, especially in adventitia, by L‐NAME/Ang II treatment in control mice. Infiltration of macrophages was significantly decreased in the aorta of MyPHD2KO mice (Figure 5C and 5D). This result was confirmed by a reduction of *F4/80* mRNA level in the aorta of MyPHD2KO mice (Figure 5E). Expression of *Tnfa, Il6, Il1b, and Mcp1*, proinflammatory genes, in the aorta was increased by L‐NAME/Ang II treatment, and they were suppressed in MyPHD2KO mice compared with control mice (Figure 5F). However, suppression of *Tnfa* was not statistically significant. Expression of *Col1a2, Col3a1, Tgfb, and Ctgf,* fibrosis‐associated genes, was increased by L‐NAME/Ang II treatment, and they were also decreased in MyPHD2KO mice compared with control mice (Figure 5G). To investigate whether these antiinflammatory and antifibrotic effects of *Phd2* deletion in macrophages were mediated by HIF up‐regulation, we administered digoxin, a drug that inhibits HIF‐α synthesis,^17^ to MyPHD2KO mice treated with L‐NAME/Ang II. Digoxin did not affect blood pressure level but mildly decreased HR (Table 3). Increased HIF‐1α and HIF‐2α levels in PMs from MyPHD2KO mice were suppressed by administration of digoxin (Figure 5H). Reduced thickening of media and adventitia in L‐NAME/Ang II‐treated MyPHD2KO mice was reversed by digoxin treatment (Figure 5A and 5B). Infiltration of macrophages and *F4/80* expression were modestly increased by digoxin treatment. However, the difference was not statistically significant (Figure 5C through 5E). Reduced cytokine expression in L‐NAME/Ang II‐treated MyPHD2KO mice was not affected by cotreatment with digoxin (Figure 5F). However, the reduced expression of fibrosis‐associated genes such as *collagen* and *Tgfb* in MyPHD2KO mice treated with L‐NAME/Ang II was reversed by digoxin (Figure 5G).

**Table 3.** Body Weight and Hemodynamic Parameters

	Control	MyPHD2KO	Control+L/A	MyPHD2KO+L/A	MyPHD2KO+L/A+Digoxin
BW, g	27.3±0.5	25.6±0.4	25.7±0.6	25.3±0.7	25.2±0.3
HW/BW, mg/g	4.3±0.1	4.4±0.1	5.7±0.1*	5.2±0.2†	5.8±0.1*,‡
HW/TL, mg/mm	6.2±0.1	6.1±0.1	7.8±0.2*	6.8±0.3†	7.7±0.1*,‡
SBP, mm Hg	98±2.0	100±2.3	139±2.0*	138±1.9*	131±2.1*
HR, bpm	506±11	502±10	563±12*	560±23*	511±9§

Data are expressed as mean±SEM. L/A indicates L‐NAME+Angiotensin II; BW, body weight; HW, heart weight; TL, tibia length; SBP, systolic blood pressure; HR, heart rate; SEM, standard error of the mean.

* *P*<0.05 vs control,

^†^ *P*<0.05 vs Control+L/A.

^‡^ *P*<0.05,

^§^ *P*<0.01 vs MyPHD2KO+L/A n=5.

### Attenuation of Cardiac Hypertrophy and Fibrosis in MyPHD2KO Mice

**Figure 6.**
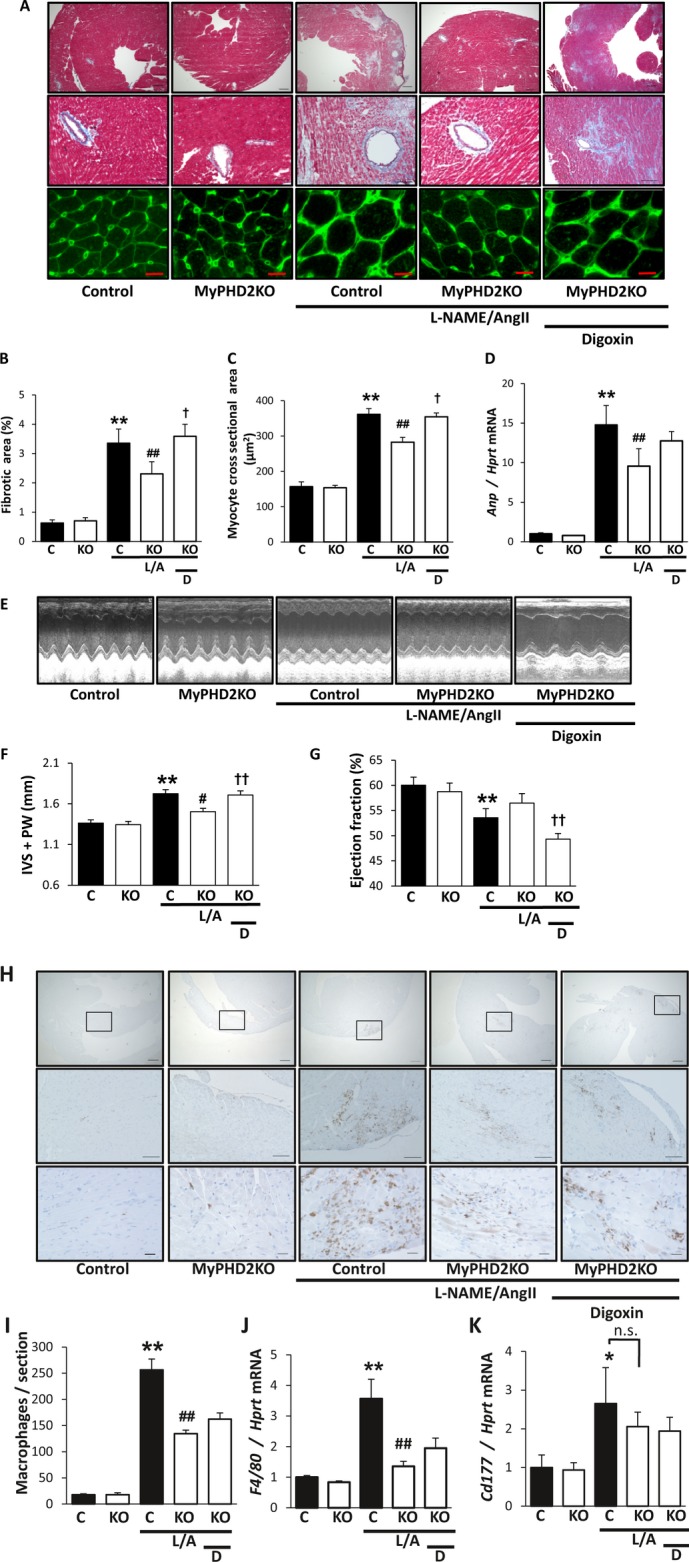
Attenuation of cardiac hypertrophy and fibrosis in MyPHD2KO mice. A, Representative photographs of Masson Trichrome staining (top and middle) and WGA staining (bottom) of the heart are shown. Scale bar: 300 μm (top), 100 μm (middle), and 10 μm (bottom). B, Summary results of fibrotic area are shown. n=8 (C), 10 (KO), 11 (C+L/A), 12 (KO+L/A), 5 (KO+L/A+D). C, Summary results of myocyte cross‐sectional area are shown. n=5. D, *Anp* expression in heart was analyzed by RT‐qPCR. n=8 (C), 8 (KO), 9 (C+L/A), 10 (KO+L/A), 5 (KO+L/A+D). E, Representative photographs of M‐mode transthoracic echocardiographic analyses are shown. F and G, Summary results of the thickness of interventricular septum (IVS) plus posterior wall (PW) (F), and ejection fraction (G) are shown. n=5. H, Immunohistochemical analysis of heart for Mac‐2 staining is shown. Scale bar: 300 μm (top), 100 μm (middle), and 50 μm (bottom). I, Summary results of the number of Mac‐2 positive cells in heart section are shown. n=3 (C), 3 (KO), 5 (C+L/A), 5 (KO+L/A), 5 (KO+L/A+D). J and K, Expression of cardiac *F4/80 *mRNA and *CD177 *mRNA was analyzed by RT‐qPCR. n=8 (C), 8 (KO), 9 (C+L/A), 10 (KO+L/A), 5 (KO+L/A+D). *P<0.05, **P<0.01 vs C, #*P*<0.05, ##*P*<0.01 vs C+L/A, †*P*<0.05, ††*P*<0.01 vs KO+L/A. WGA indicates wheat germ agglutinin; C, control, KO, MyPHD2KO; L/A, L‐NAME/Ang II; D, digoxin; *Anp*, atrial natriuretic peptide; RT‐qPCR, real‐time reverse transcription‐quantitative polymerase chain reaction; Hprt, hypoxanthine phosphoribosyl‐transferase.

The ratio of heart weight (HW) to body weight (BW) was significantly increased by administration of L‐NAME/Ang II (Table 3). The ratio of HW/BW and HW/Tibia length (TL) was smaller in MyPHD2KO mice compared with control mice. Heart sections stained with Masson Trichrome revealed increased interstitial fibrosis in both groups of mice receiving L‐NAME/Ang II, but the interstitial fibrosis was significantly attenuated in MyPHD2KO mice compared with control mice (Figure 6A and 6B). Cross‐sectional areas of myocytes were enlarged and *Anp* expression was increased by L‐NAME/Ang II in control mice, which was attenuated in MyPHD2KO mice (Figure 6A, 6C, and 6D). Echocardiographic analysis revealed that wall thickness of interventricular septum and posterior wall, FS, and EF were comparable between control and MyPHD2KO mice at baseline. Wall thickness was increased and FS and EF were suppressed after L‐NAME/Ang II treatment in control mice, which was not observed in MyPHD2KO mice (Figure 6E through 6G, Table 4). Infiltration of macrophages into the heart was increased by L‐NAME/Ang II treatment. Infiltration of macrophages was attenuated in MyPHD2KO mice (Figure 6H and 6I).

**Table 4.** Echocardiographic Characteristics

	Control	MyPHD2KO	Control+L/A	MyPHD2KO+L/A	MyPHD2KO+L/A+Digoxin
IVS, mm	0.70±0.03	0.68±0.02	0.88±0.03†	0.76±0.03‡	0.86±0.03*,§
PW, mm	0.65±0.04	0.66±0.04	0.84±0.04†	0.75±0.03‡	0.85±0.03*,§
FS, %	31.4±1.1	30.5±1.2	27.2±1.2†	29.0±0.9	24.5±0.6†,§
EF, %	60.0±1.6	58.7±1.7	53.5±1.8†	56.6±1.9	49.3±1.1†,§

Data are expressed as mean±SEM. L/A indicates L‐NAME+Angiotensin II; IVS, Interventricular septum; PW, posterior wall; FS, Fractional shortening; EF, Ejection fraction; SEM, standard error of the mean.

* *P*<0.05,

^†^ *P*<0.01 vs control,

^‡^ *P*<0.01 vs Control+L/A,

^§^ *P*<0.01 vs MyPHD2KO+L/A. n=5.

Attenuated cardiac hypertrophy, fibrosis, and *Anp* expression in MyPHD2KO mice were reversed and EF was reduced by administration of digoxin (Figure 6A through 6G). Although digoxin modestly increased macrophage infiltration in L‐NAME/Ang II‐treated MyPHD2KO mice, the difference was not statistically significant (Figure 6H and 6I), which was consistent with the data on *F4/80* expression in the heart (Figure 6J). Although we could not detect Gr‐1‐positive granulocytes by immunohistochemical analysis (data not shown), expression of *CD177*, a neutrophil specific antigen, was increased in L‐NAME/Ang II‐treated control mice. However, *CD177* expression was comparable between L‐NAME/Ang II‐treated control and MyPHD2KO mice (Figure 6K). Digoxin treatment did not affect *CD177* expression level.

**Figure 7.**
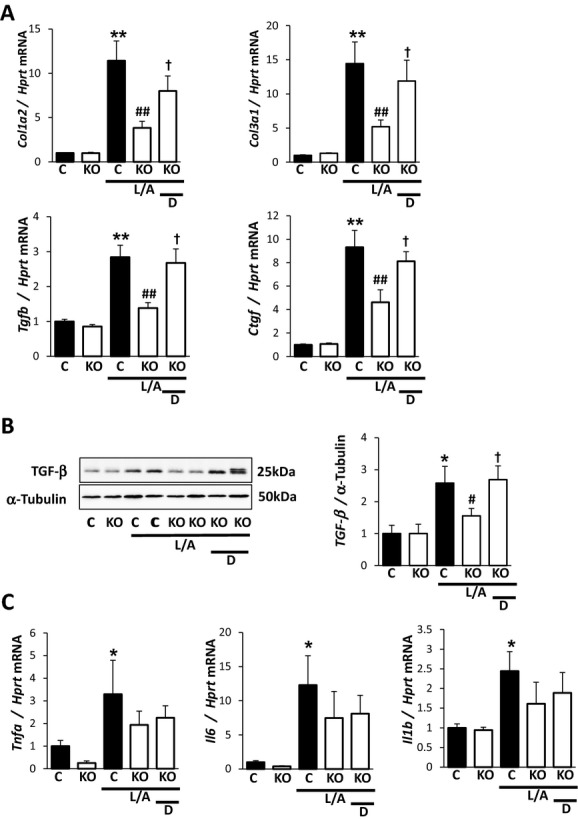
Cardiac proinflammatory and fibrosis‐associated genes were reduced in MyPHD2KO mice. A, Fibrosis‐associated gene expression in the heart was analyzed by RT‐qPCR. n=8 (C), 8 (KO), 9 (C+L/A), 10 (KO+L/A), 5 (KO+L/A+D). B, Western blot for TGF‐β in cardiac tissue is shown. The bar graph indicates the expression ratio of TGF‐β to α‐Tubulin, n=4. C, Proinflammatory gene expression in the heart was analyzed by RT‐qPCR. n=8 (C), 8 (KO), 9 (C+L/A), 10 (KO+L/A), 5 (KO+L/A+D). *P<0.05, **P<0.01 vs C, #*P*<0.05, ##*P*<0.01 vs C+L/A, †*P*<0.05 vs KO L/A. RT‐qPCR indicates real‐time reverse transcription‐quantitative polymerase chain reaction; C, control; KO, MyPHD2KO; L/A, L‐NAME/Ang II; D, digoxin; TGF‐β, transforming growth factor‐β; Col, collagen; Hprt, hypoxanthine phosphoribosyl‐transferase; Tgf, transforming growth factor; Ctgf, connective tissue growth factor; Tnf, tumor necrosis factor; Il, interleukin.

Cardiac expression of fibrosis‐associated genes (*Col1a2, Col3a1, Tgfb, and Ctgf)* was increased by administration of L‐NAME/Ang II in control mice, and they were reduced in MyPHD2KO mice (Figure 7A). The suppression was reversed by digoxin treatment, which suggests that suppression of these genes may be dependent on HIF. Protein level of TGF‐β was also increased in L‐NAME/Ang II‐treated control mice, which was not observed in MyPHD2KO mice (Figure 7B). The suppression of TGF‐β protein was reversed by cotreatment with digoxin, which is consistent with the data by RT‐qPCR analysis.

Cardiac expression of proinflammatory cytokines (*Tnfα, Il6, and Il1b*) was enhanced in L‐NAME/Ang II‐treated control mice, which was attenuated in MyPHD2KO mice with borderline significance (Figure 7C). The expression of proinflammatory cytokines was not affected by digoxin treatment.

## Discussion

In the present study, we showed that *Phd2* deletion in myeloid lineage attenuates L‐NAME/Ang II‐induced vascular and cardiac remodeling including cellular hypertrophy and fibrosis. Migration of PHD2‐deficient macrophages was attenuated and macrophage infiltration into the heart and aortic adventitia was decreased in MyPHD2KO mice after L‐NAME/Ang II treatment. M1 markers of macrophages were generally suppressed in PHD2‐deficient macrophages. However, clear M2 polarization was not observed at baseline and after L‐NAME/Ang II treatment. This study suggests that PHD2 plays an important role in macrophage migration and expression of proinflammatory and profibrotic genes that induce hypertensive cardiovascular remodeling.

Although the detailed mechanism of an antihypertrophic and antifibrotic effect of *Phd2* deletion is still elusive, down‐regulation of TGF‐β in macrophages of MyPHD2KO mice may play a role. TGF‐β is a pleiotropic cytokine and is involved in both cardiac hypertrophy and fibrosis.^22–23^ Overexpression of TGF‐β in the heart induces significant cardiac hypertrophy and fibrosis and blocking of TGF‐β activity ameliorates myocardial fibrosis and diastolic dysfunction.^24^ TGF‐β modulates fibroblast phenotype and function. TGF‐β induces myofibroblasts differentiation and synthesis of extracellular matrix protein.^25–26^ TGF‐β also enhances fibrosis through induction of CTGF, another fibrogenic mediator that was suppressed in MyPHD2KO mice. A recent study showed that endothelium‐specific deletion of HIF‐1α resulted in increased TGF‐β signaling.^27^ The heart showed excessive myocardial hypertrophy and fibrosis after transverse aortic constriction in these mice. The present study suggests that accumulation of HIF in PHD2‐deficient macrophages may suppress TGF‐β1 production. In terms of TGF‐β regulation, a recent study showed that PHD2 knockdown in tumor cells suppressed tumor growth via the antiproliferative effects of TGF‐β upregulation.^28^ Further study is needed to clarify the role of PHD2/HIF in TGF‐β regulation.

Concomitant administration of digoxin to inhibit HIFα synthesis^17^ reversed the attenuated hypertrophy and fibrosis of the heart and aorta in MyPHD2KO mice. Decreased cardiac TGF‐β and CTGF expression in MyPHD2KO mice was also reversed by digoxin. These data suggest that suppression of fibrosis‐associated gene expression is HIF‐dependent. However, reduced cytokine expression in MyPHD2KO mice treated with L‐NAME/Ang II was not reversed by HIF suppression by digoxin. Therefore, attenuation of cytokine expression in MyPHD2KO mice may be HIF‐independent. Our previous study suggests that *Phd2* down‐regulation inhibits NF‐κB,^16^ and NF‐κB activity showed a trend toward a decrease in PM from MyPHD2KO mice compared with control mice (Figure 2D). Therefore, decreased NF‐κB transcriptional activity may play a role in the down‐regulation of proinflammatory cytokine expression in PHD2‐deficient mice independently of the HIF pathway.

Inflammatory cytokines play an important role in the development of heart failure.^29^ Although L‐NAME/Ang II‐induced cytokine production was attenuated in MyPHD2KO mice, it is not clear whether the attenuation merely reflects suppression of cytokine expression in each macrophage or reduction of cytokine expression from the heart or aorta. However, our preliminary data on gene expression in cardiac tissue obtained by laser capture microdissection suggest no significant change in cytokine expression among the 5 groups in myocytes (data not shown). Therefore the changes of cytokine expression in the heart and aorta in MyPHD2KO mice may be mainly ascribed to infiltrated macrophages. However, because gene expression of fibrosis‐associated proteins was not different between PM from control mice and PM from MyPHD2KO mice (Figure 4C), we could not exclude the possible contribution of cardiac myocytes and fibroblasts to the production of fibrosis‐associated proteins. Because cytokine expression in PM was decreased and macrophage infiltration was attenuated in MyPHD2KO mice, both mechanisms seem to contribute to the reduction of cytokine expression in the heart and aorta.

A protective role of HIF‐1α is reported in a model of unilateral ureteral obstruction (UUO). Kobayashi et al showed that myeloid lineage‐specific stabilization of HIF‐1α and HIF‐2α by deletion of *von Hippel Lindau*, a substrate recognition subunit of the ubiquitin ligase complex for HIF‐α proteasomal degradation, attenuated renal damage by UUO, which was characterized by a decrease in macrophage infiltration.^15^ In addition, myeloid‐specific deletion of *HIF‐1α* and *HIF‐2α* enhanced macrophage infiltration. However, *Collagen‐1a* mRNA level or accumulation of extracellular matrix was not changed in both models. Our study showed that both HIF‐1α and HIF‐2α were accumulated in PM and that fibrosis of the heart and aorta was attenuated in association with a decrease in macrophage infiltration. Although the reason for this discrepancy between the UUO model and ours is not immediately clear, it may be due to the difference of the model examined. Alternatively, the difference may suggest that other unknown substrates of PHD2 play a role in fibrosis. In addition to HIF‐α, several studies have identified novel PHD2 substrates such as Sprouty 2 and β‐arrestin.^30–31^ We examined expression level of Sprouty 2 and β‐arrestin in PM from control and MyPHD2KO mice. However, we did not find any difference in the expression level of Sprouty 2 or β‐arrestin at baseline and after L‐NAME/Ang II treatment (data not shown). Further study is needed to clarify gene regulation by PHD2.

A growing body of evidence suggests that the function of macrophages depends on their activation or polarization state, designated classically activated (M1) and alternatively activated (M2) macrophages. PHD2‐deficient macrophages shows reduction of M1 macrophage markers, which is consistent with previous results that examined macrophage from *Phd2* hetero knockout mice.^32^ M1 macrophages are generally associated with inflammation. Although an obvious shift to the M2 phenotype was not observed at baseline and after L‐NAME/Ang II treatment, reduction of M1 markers may play a role in the reduction of hypertrophy and fibrosis in MyPHD2KO mice. However, it is reasonable to assume that the gene expression profile is different between PM and tissue‐infiltrated macrophages. A future study that examines gene expression of infiltrated macrophages and the interaction between infiltrated macrophages and somatic cells such as myocytes and vascular SMCs is warranted.

Another question that is not addressed in this study is the mechanism for less macrophage infiltration in L‐NAME/Ang II treated MyPHD2KO mice. Because migration of PM from MyPHD2KO mice toward MCP‐1 was reduced, it is suggested that the recruitment of macrophages is attenuated by *Phd2* deletion. However we could not exclude the possibility that macrophage apoptosis is increased during or after recruitment into the tissue. We also failed to show the role of neutrophils that also express lysozyme M.^33^ We barely found granulocyte infiltration in the heart and aorta in all groups by immunostaining with a Gr‐1 antibody. However, RT‐qPCR analysis revealed an increase in the cardiac expression of *CD177*, a neutrophil‐specific antigen, in L‐NAME/Ang II‐treated control and MyPHD2KO mice to the same extent (Figure 6K). These results suggest that deletion of *Phd2* in neutrophils may play a minor role in the attenuation of cardiovascular remodeling induced by L‐NAME/Ang II. Consistent with this idea, a recent study showed that macrophages rather than neutrophils play a critical role in Ang II‐induced vascular dysfunction and arterial hypertension.^33^

There are several limitations in the present study. The first is that although previous studies^17,27^ and the present study showed that digoxin inhibits HIF‐α synthesis, we could not exclude the possibility of nonspecific effects of digoxin on other tissues, in particular cardiac myoctyes. Because digoxin itself is expected to have a mild cardiotonic effect, we assume that HIF‐α down‐regulation in macrophages by digoxin is detrimental to cardiac function in this model. The second point is that the sample sizes are relatively small in the present study, which may explain why some statistical tests failed to reach significance.

Several PHD inhibitors are under development for clinical use as a treatment for ischemic disorders^34^ and anemia.^35^ It is not clear whether PHD inhibitors attenuate hypertensive cardiovascular remodeling. Our previous study showed that cobalt chloride, a nonspecific PHD inhibitor, attenuated perivascular fibrosis of the heart induced by Ang II. Therefore, it is possible that PHD inhibitors may be useful not only for the treatment for anemia but also for cardiovascular remodeling induced by high blood pressure.

In summary, we provided convincing evidence that PHD2 in myeloid lineage cells play a critical role in high blood pressure‐induced hypertrophy and fibrosis. PHD inhibition may be a novel of therapeutic strategy for cardiovascular diseases.
